# Nexplanon Subdermal Implant: Assessment of Sexual Profile, Metabolism, and Bleeding in a Cohort of Italian Women

**DOI:** 10.1155/2019/3726957

**Published:** 2019-01-31

**Authors:** Maurizio Guida, Manuela Farris, Carmen Imma Aquino, Elena Rosato, Lucio M. A. Cipullo, Carlo Bastianelli

**Affiliations:** ^1^Department of Obstetrics and Gynecology, School of Medicine, University of Salerno, Salerno, Italy; ^2^Department of Maternal and Child Health and Urology, Sapienza University of Rome, Italy; ^3^AIED, Rome, Italy

## Abstract

**Objectives:**

To evaluate the impact on metabolism, bleeding, and sexual function of Nexplanon, a subdermal implant.

**Study Design:**

We recruited women (*n*=101) receiving the Nexplanon implant at two university centers in Italy between 2011 and 2016 into this prospective, observational, multicenter research trial. Participants completed the Interview for Ratings of Sexual Function (IRSF) and the Female Sexual Function Index (FSFI) questionnaires before and 3 and 6 months after the implant was inserted. In addition, all blood parameters were assessed at these visits. All women were given a menstrual diary card and a pictorial blood assessment chart to record daily any vaginal bleeding.

**Results:**

The studied metabolic parameters remained in the normal range, showing no alarming modifications: minimal statistical reductions (in aspartate aminotransferase, alanine aminotransferase, total cholesterol, triglycerides, and activated partial thromboplastin time) and increases (in glucose and prothrombin activity) were observed. Changes in IRSF score over 6 months showed a significant increase in pleasure, personal initiative, orgasm, intensity of orgasm, and satisfaction, and a significant decrease in anxiety and discomfort. Mean Body Mass Index decreased, and the weekly frequency of sexual intercourse increased.

**Conclusions:**

Nexplanon showed not only a lower metabolic and bleeding impact, but also important positive effects on sexual function. It expands the range of possibilities for women, 38 and couples, in the modern concepts of sexual and reproductive wellbeing.

## 1. Introduction

Nexplanon is a single-rod subdermal contraceptive implant containing a total of 68 mg of etonogestrel (a progestin being the active metabolite of desogestrel derived from the 19- nortestosterone), which is released daily at low doses (25–70 *μ*g) through a rate-limiting membrane, allowing a contraceptive effect lasting up to 3 years [1]. Nexplanon is an advanced version of Implanon, differing from the latter by having an easier insertion technique, using a next-generation applicator (Merck & Co, Whitehouse Station, NJ, USA), and for its detectability on X-ray imaging and computed tomography [2].

Influence of hormonal contraceptives on sexual life is not frequently reported. Data published to date are contrasting and refer to the combined short-acting hormonal oral [3, 4] and vaginal contraceptives [5, 6], while studies on the influence of subdermal contraception are few, and not specifically focused on female sexual function [7–9].

Long-acting reversible contraceptive (LARC), specifically subdermal contraceptives, has become of increased interest for women of all ages, because they are easy to use and discreet and have a minimum impact on social/family life [10–13]. Nexplanon has the same advantages in terms of compliance that all LARC hold: women no longer need daily reminders to take the “pill” (oral contraceptive) or weekly or monthly replacement of the patch or the vaginal ring, respectively [13].

The aim of our study was to investigate the complete profile of women using Nexplanon, analyzing possible changes in metabolic parameters, the discomfort from initial spotting/bleeding associated with this contraceptive method, and—for the first time—the quality of sexual life through a visual analog scale (VAS) and the Female Sexual Function Index questionnaire.

## 2. Materials and Methods

This prospective, observational, multicenter research trial was performed at the Department of Obstetrics and Gynecology, University of Salerno and the former Department of Obstetrical Gynecological Sciences and Urological Sciences, now Department of Maternal and Child Health and Urology, Sapienza University of Rome, Rome (Italy).

Women attending our centers between 2011 and 2016, for regular gynaecologic assessments or with a contraceptive request or for periodic check-ups at our Center for Birth Control, were invited to enrol in the study. During this period, the Interview for Ratings of Sexual Function (IRSF) and the Female Sexual Function Index (FSFI) questionnaires were routinely administered to all women requesting a contraceptive, to obtain a database of all contraceptive methods. In addition, blood parameters were assessed for all women attending the clinics.

Women were included in the study if they had a regular menstrual cycle and body mass index (BMI) of 19.5–26.6 kg/m2 and were sexually active. Exclusion criteria were reported venous thromboembolic disorders, known or suspected malignancy, severe liver disease, and undiagnosed vaginal bleeding. Additional exclusion criteria for this analysis were hypersensitivity to the active ingredient or to any other components of Nexplanon, or the use of drugs that could affect its pharmacokinetics

The study protocol was approved by the Ethical Review Committees of both Universities and was carried out following the principles of the Declaration of Helsinki. Confidentiality of the participants was maintained during data analysis. All participants provided written, informed consent before entry into the study and after the research protocol had been verbally explained to them in detail.

A detailed medical history and a gynaecological examination including vaginal swab and Papanicolau test were performed at screening, and blood pressure, BMI, and blood parameters (glucose, blood urea nitrogen, creatinine, aspartate aminotransferase [AST], alanine aminotransferase [ALT], cholesterol, triglyceride, antithrombin III, prothrombin time, activated partial thromboplastin time, fibrinogen, and platelets) were assessed for all women.

The Nexplanon subdermal implant was inserted within 5 days of the onset of menstruation by trained physicians.

Sexual profile was assessed using the IRSF, a structured 11-item interview, in which each item presents a VAS of 100 mm (with a range of 0–100) [5] and the FSFI, a multidimensional validated questionnaire for assessing female sexual function [14]. The IRSF and the FSFI were administered to participants before the implant use and 3 and 6 months after its insertion.

To respect privacy and avoid the bias that may result from a face-to-face approach, the questionnaires are designed to be self-administered. Participants completed the questionnaires by themselves after each part had been explained by a trained physician.

The effects of contraception on sexual profile of participants were calculated as difference in VAS score (ΔVAS) before the implant (VAS0) and after 3 months (VAS3) and 6 months (VAS6) of insertion of the implant. The same was done for blood parameters (ΔMET).

All women were given a menstrual diary card to record daily any vaginal bleeding. Bleeding and spotting days were assessed using the World Health Organization reference period developed by Belsey and Farley [15]. All women were instructed on the correct distinction between bleeding (use of more than one sanitary pad per day) and spotting (only one sanitary pad needed per day). Bleeding was defined as unchanged if the characteristics were identical to those present before implant insertion. The diary cards were evaluated at 3 months and at 6 months. Quantity of menstrual blood flow was assessed through the Menstrual Pictorial Blood Assessment Chart by Higham [16]

Age, BMI, duration of relationship, and weekly frequency of sexual intercourse were reported as mean and standard deviation (SD). The means of BMI and weekly frequency of 1 sexual relation were compared at baseline (T0) and after 6 months of use (T1), using the t-test for paired samples.

The Mann-Whitney test for independent variables was used for statistical evaluation of ΔVAS1 (VAS3–VAS0), ΔVAS2 (VAS6–VAS0), and ΔVAS3 (VAS6–VAS3) and the corresponding differences between the individual metabolic parameters. The method calculates the sum of ranks for each group and compares this with what would be expected by chance. The statistical significance was set at p<.05.

## 3. Results

**Figure 1:**
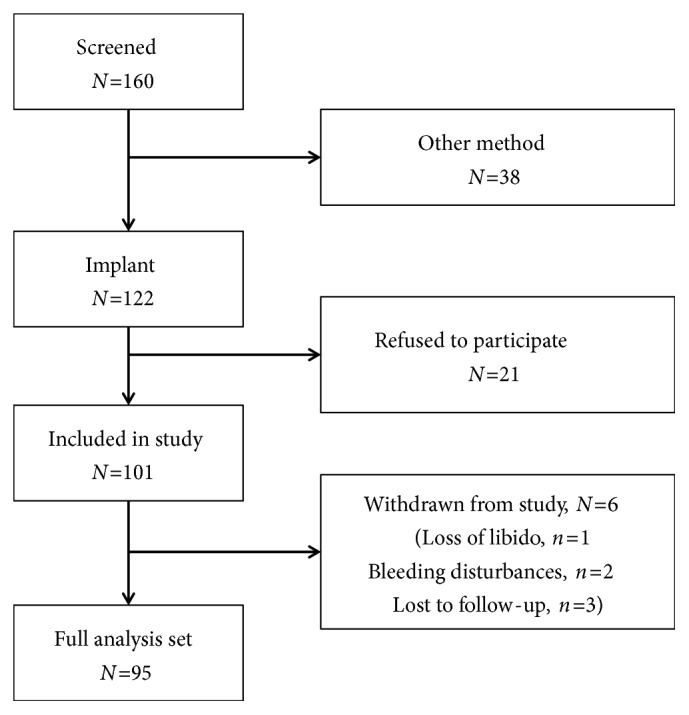
Study flowchart.

Of 160 women screened, 122 were eligible and 101 agreed to participate in the study. Six women were excluded from the final dataset: one discontinued the use of contraception because of signalled decreased libido, two discontinued for irregular bleeding, and three were lost to follow-up (Figure 1). In the dataset of subjects analyzed, the implant was retained for the entire 6-month period of evaluation.

Participants' characteristics are reported in Table 1. Mean age (± SD) of the women was 28.8±6.8 years, mean BMI was 23.4±2.5 kg/m^2^, and average length of relationship was 7.7±5.9 years.

A reduction in mean BMI was observed after 6 months, as was an increase in weekly frequency of sexual intercourse (T0–T1). The mean change (± SD) in BMI was 0.5±0.9 (p=.013) and in frequency of sexual intercourse −1± 1.5 (p=.003).

**Figure 2:**
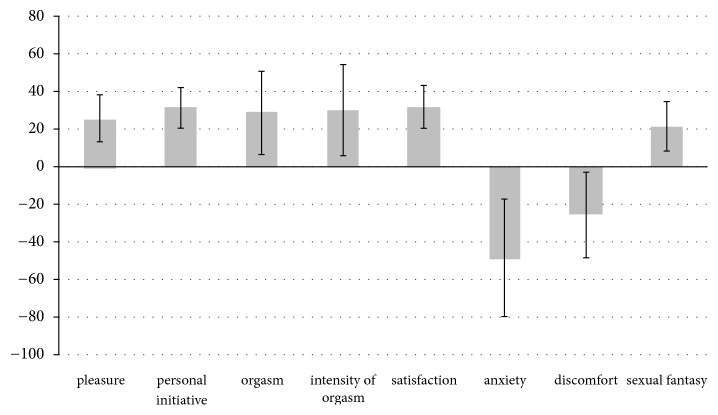
Effects on sexual function in terms of ΔVAS at 6 months (VAS6) after insertion. The evaluation was performed for each item investigated through the Interview for Ratings of Sexual Function (IRSF) questionnaire.

Sexual profile showed a significant increase in some positive parameters of the IRSF (pleasure, personal initiative, orgasm, intensity of orgasm, and satisfaction) and a significant decrease in some negative parameters (anxiety and discomfort, Figure 2). In particular, at 3 months after implant insertion, analysis of ΔVAS1 showed an increase in orgasms, orgasm intensity, and satisfaction, and a decrease in anxiety; at 6 months, ΔVAS2 demonstrated an increase in pleasure, personal initiative, orgasm, and satisfaction and a reduction in anxiety and discomfort. No difference was seen in ΔVAS3 (Figure 2).

**Figure 3:**
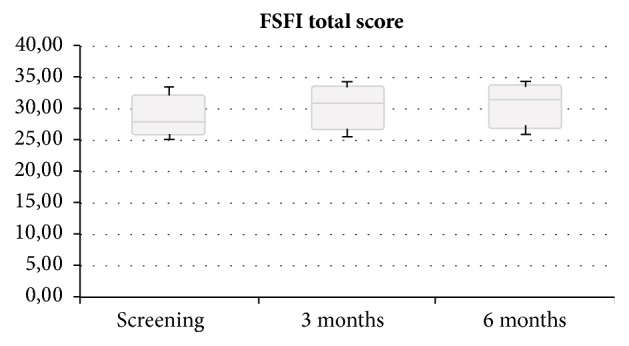
Total Female Sexual Function Index (FFSI) score at recruitment and 3 and 6 months after insertion of Nexplanon. Data are expressed as mean (minimum to maximum).

These improvements corresponded to the data obtained from the FSFI questionnaire: FSFI total score was positively correlated with the use of Nexplanon (Figure 3). Before the implant was inserted, total score was 27.8 (range, 25.1–33.4). This increased to 30.8 (25.4–34.2) after 3 months and 31.4 (25.7–34.2) after 6 months.

**Figure 4:**
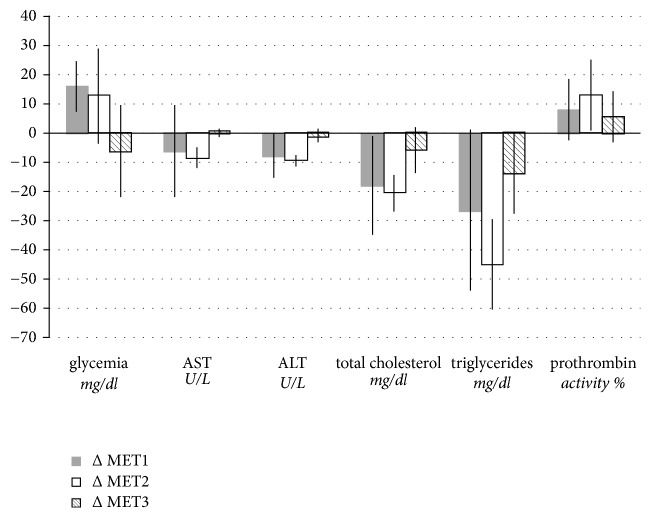
Metabolic parameters were evaluated before implant insertion (MET0) and 3 months (MET3) and 6 months (MET6) after implant. For each parameter measured, we calculated the difference (ΔMET) at three different times: ΔMET1 = MET3–MET0; ΔMET2 = MET6–MET0; ΔMET3 = MET6–MET3. Data are expressed as mean ± SD.

Overall, metabolic parameters remained within the normal range, with a minimal decrease in AST, ALT, total cholesterol, triglycerides, and activated partial thromboplastin time values and an increase in glycemia and prothrombin activity (Figure 4). In detail, at 3 months after implant insertion, we found an increase in glycemia and prothrombin activity and a reduction in AST, ALT, total cholesterol, and triglycerides; after 6 months of implant, we found a reduction in AST, ALT, total cholesterol, and triglycerides and an increase in prothrombin activity. All changes were nonsignificant and within the normal range.

Between first and second control there was a reduction in triglycerides that was shown to be statistically significant (p=.04).

When bleeding diaries were evaluated, a reduction in menstrual flow quantity was observed in 50% of women. Amenorrhea was reported by 9% at 3 months and increased to 23% at 156 6 months. The proportion reporting infrequent bleeding also increased, whereas irregular and prolonged bleeding decreased over this time (Table 2).

## 4. Discussion

The influence of the subcutaneous contraceptive Nexplanon on the sexual profile is currently not well reported in literature and, to the best of our knowledge, ours is one of the most complete studies to analyze libido in users of this long-acting contraceptive through both a VAS and FSFI score.

Women reported an increase in frequency and quality of coitus and documented an increase in pleasure, satisfaction, frequency, and intensity of orgasm. Participants also reported an increase in sexual desire, which was confirmed by a statistically significant increase in personal initiative/drive.

The positive effect of this type of contraception was further supported by the reduction in anxiety and discomfort, and women experienced intercourse with greater freedom and sense of safety.

Our data show that the use of Nexplanon involves a more confident psychological predisposition to sexual behavior in women. We do not know if these positive effects could also be related to the high contraceptive efficacy perceived and reported [17–19].

The few published studies on the use of Nexplanon implant and sexuality are on line with our data. Di Carlo and its group [20] showed that the implant seems to have a positive impact on quality of life after the first three months of therapy without any negative effects on libido and on sexual function.

Moreover, when the implant was compared to other types of contraceptives women who used the subcutaneous implant had higher scores on the McCoy female sexuality questionnaire and higher levels of androstenedione [9, 13].

Once the issue of sexuality was addressed utilizing other LARC, progestin only contraceptives, namely, the levonorgestrel intrauterine system (LNG-IUS), a positive effect on quality of life and sexuality has been reported [21].

In a multicenter cross-sectional study Enzlin and coll. concluded that women using a LNG-IUS do not differ from those wearing a Cu-IUD with regard to psychological and sexual functioning [22].

Also, the decreased body weight and BMI in users of Nexplanon could have been a factor which improved sexual life because a better body image and confidence can of course have psychosocial and relational consequences.

Changes in menstrual patterns were not regarded to be of relevance by the women, and the observed metabolic effects were not of concern to women's health.

As the influence of the subcutaneous contraceptive Nexplanon on the sexual profile is currently not well reported in literature, to the best of our knowledge ours is one of the most complete studies to analyze libido in users of this long-acting contraceptive through both a VAS and FSFI score.

Limits of our study are the small sample size, and given the importance of this topic and its potential effects on the fertile population, a future study with a larger number of observed cases could give more detailed information and confirmation of our results.

Sexual acceptability should receive more attention in both contraceptive research and counseling. Women's perceptions of how their method affects their sex life were associated with contraceptive continuation over time [23].

## Figures and Tables

**Table 1:** Demographic characteristics of women who completed the study (*N*=95).

**Characteristic**	**n**	%
Age, years		
<18	5	5.3
18–22	28	29.4
23–27	45	47.4
28–32	11	11.6
>32	6	6.3
Nationality		
Italian	79	83.1
Other	16	16.9
Marital status		
Unmarried	63	66.3
Married	28	29.5
Divorced	4	4.2
Employment		
Student	42	44.2
Employed	22	23.2
Housewife	18	18.9
Unemployed	13	13.7
Previous contraceptive use		
Yes	86	90.5
No	9	9.5
Method used		
Condom	15	15.8
Combined hormonal contraceptive	27	28.4
Combined contraceptive methods (e.g., COC + condom)	49	51.6
Other methods (e.g., IUD)	4	4.2

**Table 2:** Menstrual patterns at 3 months and 6 months.

	**3 months**		**6 months**		**p**
	n	%	n	%	
Not bleeding (amenorrhea)	9	9.5	22	23.2	.03
Infrequent bleeding (<2 episodes)	19	20.0	31	32.6	NS
Frequent bleeding (4 episodes)	16	16.8	9	9.5	NS
Irregular bleeding, range of lengths of bleeding-free intervals exceeding 17 days	31	32.6	16	16.8	.03
Prolonged bleeding (>10 days)	13	13.7	5	5.3	NS
Normal menstrual pattern	7	7.4	12	12.6	NS

NS= not significant.

## Data Availability

The data used to support the findings of this study are available from the corresponding author upon request.
